# Quality evaluation of *Lycium barbarum* (wolfberry) from different regions in China based on polysaccharide structure, yield and bioactivities

**DOI:** 10.1186/s13020-019-0273-6

**Published:** 2019-11-08

**Authors:** Ying Wang, Hongyu Jin, Xiaoxv Dong, Shuang Yang, Shuangcheng Ma, Jian Ni

**Affiliations:** 1 School of Chinese Materia Medica, Beijing University of Chinese Medicine, No.11 North 3rd Ring East Road, Chaoyang District, Beijing, 100102 China; 2 Institute for Control of Chinese Traditional Medicine and Ethnic Medicine (ICCTMEM), National Institutes for Food and Drug Control (NIFDC), No. 2 Tiantan Xili, Dongcheng District, Beijing, 100050 China; 3 School of Medicine and Pharmacy, Ocean University of China, Qingdao, 266003 China

**Keywords:** *Lycium barbarum* L., Polysaccharide, Quality evaluation, Antioxidative activity, Immunomodulatory activity

## Abstract

**Background:**

*Lycium barbarum* (wolfberry) has been widely cultivated in China, particularly in northwest regions. However, the fruit size and taste of *L. barbarum* from different habitats are quite different. Traditionally, only the fruit of *L. barbarum* produced in Ningxia province is recorded as an authentic herb, although the detailed mechanism responsible for this remains obscure. Polysaccharides are considered major active ingredients in *L. barbarum* which is crucial for its quality evaluation.

**Methods:**

In this study, we assessed the yield, monosaccharide composition, molecular weight, and conformation of *L. barbarum* polysaccharides (LBPs) collected from different regions of China. The antioxidant and immune activities of LBPs were also determined as its quality indicator.

**Results:**

Our results showed that the similarity values of monosaccharide composition were larger than 0.926, and the *M*w of the two fractions (peaks 1–2) in LBPs were ranging from 1.36 × 10^6^ to 2.01 × 10^6^ (peak 1), and 6.85 × 10^4^ to 10.30 × 10^4^ (peak 2) which indicated that the structure of LBPs were similar. In addition, results showed that there was no significant difference in antioxidant and immune activities of nine LBPs from different regions. However, the yield of LBPs from Qinghai Province (low atmospheric temperature, high altitude) was significantly lower (*p* < 0.05) than those collected from Xinjiang and Ningxia province.

**Conclusions:**

These data suggested that the *L. barbarum* produced in Ningxia and Xinjiang maybe more suitable as materials for medicines and functional foods. This study also provides a reference for improving the quality control standard of LBPs.

## Background

*Lycium barbarum* L., a well-known Chinese herb, is one of the medicinal and food homologous traditional Chinese medicines. *L. barbarum* products have been classified as nutraceutical foods or dietary supplements and have become popular in East Asia, Europe, and North America [1]. Generally, the polysaccharide is the main bioactive ingredient in *L. barbarum*, which exerts various effects, including immunomodulation, antioxidant, anti-hypertension, and anticancer activities [2]. In China, *L. barbarum* has been widely cultivated, particularly, in northwest regions, such as Qinghai, Xinjiang, Ningxia, and Gansu. However, the fruit size and taste of *L. barbarum* from different habitats are considerably different. Traditionally, only the fruit of *L. barbarum* produced in Ningxia is recorded as an authentic (Daodi) herb in China. Whether differences in the structure and activity of *L. barbarum* polysaccharides (LBPs) from typical regions exist is crucial for its quality evaluation and related product development.

Unlike small-molecule compounds, polysaccharides are difficult to evaluate only by their content determination or a specific spectrum. In the 2015 edition of Chinese Pharmacopoeia and a few studies [3], the polysaccharide quality of *L. barbarum* was evaluated only by determination of the content of total sugars using the phenol–sulfuric acid method with glucose as a reference standard. Indeed, this method is not appropriate for quality evaluation of LBPs due to its poor specificity and inability to reflect the structural characteristics of LBPs [4]. Structural features, including sugar compositions, molecular weight, and conformation are all required for comprehensive description of the polysaccharide products. In recent studies, multiple fingerprints of LBPs have been established [5]. Researcher also compared the LBPs obtained from different locations by molecular weight (*M*w) determination [6]. The results in these studies showed that the similarity of those polysaccharides in *L. barbarum* collected from different regions of China was high. Moreover, antioxidant and immune activity of LBPs have been studied in some researches. But these reports focused on the antioxidant and immune activity of different separated fractions in LBPs [7, 8]. Xie and others evaluated and compared the effects of eight LBPs from different producing areas on macrophage function [9]. In our study, the quality of LBPs from different producing areas was comprehensively evaluated by structure, yield and activity.

In this study, nine LBPs samples of the identical varieties from different locations were extracted and analyzed to determine their structural properties, including the monosaccharide composition, *M*w and conformation. The similarity of LBPs from different regions was assessed using statistical and similarity analyses with professional software. Polysaccharides are the major bioactive compounds in *L. barbarum* by report, which have possessed the immunological adjuvant effect and antioxidant activity. To obtain deep insights into the quality of *L. barbarum* polysaccharides, their antioxidant and immune activity were also detected as a quality indicator. The antioxidant activities of LBPs were evaluated by hydroxyl and ABTS radicals scavenging assays. In addition, immune activity were evaluated by detect the effect of LBPs on NO, TNF-α, IL-6 production of macrophage. The results could serve as a scientific basis to further evaluate the quality of *L. barbarum* and will provide a reference for improving the quality control standard of LBPs production.

## Materials and methods

### Materials

In 2018, nine batches of *L. barbarum* were collected from three main producing regions of China, including Ningxia (NX), Qinghai (QH) and Xinjiang (XJ) (Table 1). The samples of *L. barbarum* were identified by Professor NanPing Zhang from the National Institute for Food and Drug Control, China. For authentication, their species were confirmed by a survey of their plants in cultivation base, and the macromorphological properties of the fruits.

**Table 1:** Chemical composition of LBPs in nine samples

Code	Origins	Protein content (%)	Total carbohydrate content (%)	Yield (%)
NX1	Zhongning, Ningxia	4.55	64.95	3.7
NX2	ZhongWei, Ningxia	5.53	68.01	3.9
NX3	Pingluo, Ningxia	4.08	69.08	4.4
Mean ± SD (n = 3)			67.4 ± 3.21	4.0 ± 0.32
QH1	Gonghe, Qinghai	4.13	65.92	1.9
QH2	Geermu, Qinghai	3.60	60.79	1.7
QH3	Dulan, Qinghai	4.20	59.05	1.6
Mean ± SD (n = 3)			61.9 ± 5.80	1.7 ± 0.07*
XJ1	Jinghe, Xinjiang	5.50	60.15	4.8
XJ2	Wulumuqi, Xinjiang	4.38	62.27	4.4
XJ3	Kuermu, Xinjiang	3.64	65.15	4.2
Mean ± SD (n = 3)			62.5 ± 4.05	4.5 ± 0.31

*p < 0.05 vs Ningxia group

d-Mannose (Man), d-ribose (Rib), l-rhamnose (Rha), d-glucuronic acid (GlcA), d-galacturonic acid (GalA), d-glucose (Glc), d-galactose (Gal), d-xylose (Xyl), and l-arabinose (Ara) were obtained from the National Institute for Food and Drug Control (China). Bovine serum albumin (BSA) was manufactured by Thermo (USA). 1-Phenyl-3-methyl-5-pyrazolone (PMP), 2,2-azino-bis-(3-ethylbenzothiazoline-6-sulfonic acid) (ABTS) and 2,2-diphenyl-1-picrylhydroxyl (DPPH) were purchased from Sigma-Aldrich Trading (St. Louis, MO, USA). Other reagents and chemicals were of analytical grade and obtained from Sinopharm Chemical Reagent Co. Ltd. (Shanghai, China).

### Preparation of LBPs

Each of the samples (5.0 g) was defatted with ether for 2 h in a Soxhlet extractor. Then, the residues were immersed twice into an 80% ethanol solution (for 1 h each time) to remove small-molecule materials and pigments. Next, the dry residues were extracted with water at 80 °C two times (for 1 h each time) [10]. After centrifugation (4000×*g* for 5 min), the supernatant was evaporated to 10.0 mL of solution under vacuum using a rotary evaporator. Next, ethanol (95%, w/v) was added to the final concentration of 80% (v/v) for precipitation and left to react overnight. The precipitate was then collected by centrifugation and successively washed with ethanol and acetone [11]. Then, the precipitate was redissolved in 10.0 mL of hot water. The 85% protein in the crude polysaccharide was removed by the Sevag’s method to avoid interference with the activity determination. Impurities in the crude polysaccharide, such as salts and particles with *M*w < 3 kDa were further removed using a ultracentrifugal filter with molecular weight cutoff of 3 kDa [6] by centrifugation (4500×*g* for 20 min). Finally, the crude polysaccharides, LBPs, were obtained by freeze-drying of the retentate of ultrafiltration. The LBPs in nine samples were prepared under the above extraction conditions for further analysis and bioactivity study. The yield of LBPs was calculated using the following equation:

$$ {\text{LBPs yield}}\% \;({\text{w}}/{\text{w}}) = \frac{\text{dried LBPs weight}}{{{\text{powder weight}}\,(5{\text{ g}})}} \times 100\% $$

### Analytical methods for composition determination

Neutral sugar levels were determined by the phenol–sulfuric acid method as d-glucose equivalents. The sulfuric acid–carbazole method was adapted to measure the uronic acid content with d-galacturonic acid used as a standard. The protein content was determined by the Bradford method using bovine serum albumin (BSA) as a standard [5].

### Determination of molecular weight and polydispersity index

The *M*w and polydispersity (*M*w/*M*n) of LBPs were measured using HPSEC-MALLS-RID methods. Each sample of 20 mg was dissolved in 2 mL of the mobile phase and then filtered through a 0.45-µm membrane. The *M*w of LBPs were established by a HPLC system (Shimadzu Company, Japan), equipped with a refractive index detector (RID), combined with a multi-angle laser light scattering detector (MALLS, DAWN HELEOS, Wyatt Technology Co., Santa Barbara, CA, USA). After optimizing the chromatographic conditions, multiple size exclusion columns Shodex SB806 (300 mm × 7.8 mm, i.d.), Shodex SB805 (300 mm × 7.8 mm, i.d.), and Shodex SB804 (300 mm × 7.5 mm, i.d.) were used to obtain a good separation efficiency. The mobile phase was 0.1% NaNO_3_ aqueous solution applied at a flow rate of 0.5 mL/min. An injection volume of 100 µL was used, and each sample was run for 80 min, and a temperature maintained at 40 °C.

### Monosaccharide composition analysis

The composition analysis of polysaccharide is an important step to control the quality and obtain basic information. Briefly, each of the LBPs (1 mg/mL) was hydrolyzed by trifluoroacetic acid (TFA, 4 mol/L) at 120 °C for 4 h, followed by complexing with PMP (0.5 mol/L) [5]. Sample solution (20 μL) was injected and analyzed by HPLC-PAD using a ZORBAX Eclipse XDB-C18 column (250 × 4.6 mm, 5 μm, Agilent, USA) and UV detection at 250 nm. The flow rate of the mobile phase consisting of acetonitrile and 0.125 mol/L KH_2_PO_4_ (v/v = 16:84, pH 6.9) was 1.0 mL/min.

### Antioxidative activity study

#### Hydroxyl radical scavenging activity

The hydroxyl radical scavenging activities of LBPs were determined using the Fenton’s reaction [12] with some modifications [13]. The hydroxyl radical was generated in the mixture of 0.8 mL of 2 mmol/L salicylic acid solution, 1 mL of 0.15 mmol/L FeSO_4_ and 1 mL of H_2_O_2_ (0.01%, v/v). After addition of 0.2 mL of the sample, the mixture was incubated at 37 °C for 60 min and the absorbance measured at 510 nm. The absorbance of the mixture (A_1_) was measured at 510 nm, the absorbance of the blank control (A_0_, water instead of sample solution) and H_2_O_2_ control (A_2_, water instead of H_2_O_2_) were measured by the same method, and V_C_ was used as positive control. The scavenging activity of the hydroxyl radical was calculated according to following formula:

$$ {\text{Scavenging activity}}\left( \% \right) = ({\text{A}}_{1} - {\text{A}}_{0} ) \, /({\text{A}}_{2} - {\text{A}}_{0} ) \times 100. $$

#### Total antioxidant capacity

The ABTS radical scavenging activities of LBPs were measured by a previously described method [14] with some modifications. The ABTS radical solution was diluted with phosphate buffer saline (pH 7.0) to an absorbance of 0.70 ± 0.02 at 734 nm. A volume of 0.2 mL of LBP solution was added to ABTS radical solution in a ratio of 1:20, and the mixture solution was incubated for 60 min in the dark. Then, the absorbance of the mixture (A_x_) and the blank control (A_0_, water instead of sample) were measured at 734 nm. V_C_ was utilized as positive control. The scavenging activity of the ABTS radical was calculated by the following equation:

$$ {\text{Scavenging activity}}\left( \% \right) = (1 - {\text{A}}_{\text{x}} /{\text{A}}_{0} ) \times 100. $$

### RAW264.7 macrophage proliferation

RAW264.7 macrophage cells in an RPMI-1640 medium containing 10% FBS were plated in a 96-well microplate (1 × 10^4^ cells/well, 100-µL volume; ATCC). The cells were incubated with 100 µL of the LBPs at different concentrations (50, 100 and 200 µg/mL) in triplicate. The cell cultures were kept in humidified atmosphere containing 5% CO_2_ at 37 °C for 72 h. Then, the WST-1 solution (20 µL) was added to the wells and the solution was further incubated for 4 h at 37 °C. The optical density was measured at 450 nm using a microplate reader (EL-800; BioTek Instruments, Winooski, VT, USA). The absorbance (A) was translated into a macrophage proliferation ratio (%) = At/Ac 100, where At and Ac are the absorbance of the test group and control group, respectively.

### Reverse transcription-polymerase chain reaction (RT-PCR)

RAW264.7 cells in logarithmic growth phase were seeded in 6-well plates with a cell density of 1 × 10^5^/mL. After 12 h of culture, the culture medium was replaced with the Dulbecco’s modified Eagle’s medium (DMEM) containing NX1, QH1, XJ1 at the concentrations of 50, 100 and 200 µg/mL, respectively, or 1 μg/mL LPS. After incubation at 37 °C for 18 h. The TRIzol reagent (Invitrogen, Carlsbad, CA, USA) was used to extract the total RNA from RAW264.7 cells according to the manufacturer’s protocol. Reverse transcriptase-generated complementary DNA encoding iNOS, IL-6 and TNF-α genes were amplified by polymerase chain reaction using specific primers. The nucleotide sequences of the primers were as follows: iNOS, 5′-TGCCACGGACGAGACGGATAC-3′(forward) and 5′-CCATTGCACAACTCTTTTCTCA-3′(reverse); IL-6,5′-CTCCCAACAGACCTGTCTATAC-3′(forward) and 5′-CCATTGCACAACTCTTTTCTCA-3′(reverse); TNF-α,5′-ATGTCTCAGCCTCTTCTCATTC-3′(forward) and 5′-GCTTCTCACTCGAATTTTGAGA-3′(reverse); GAPDH,5′-TGGCCTTCCGTGTTCCTAC-3′(forward) and 5′-GAGTTGCTGTTGAAGTCGCA-3′(reverse). Each sample was tested three times and the average of the three values was used for calculation. In a parallel experiment, glyceraldehyde-3-phosphate dehydrogenase (GAPDH) was used as an internal control.

### Statistical analysis

Each assay was performed in triplicate. The data are expressed as mean ± standard deviation (SD). Statistical differences between the different groups were assessed by Student’s *t*-test. The similarities in the HPLC chromatograms were evaluated by the software “Similarity Evaluation System for Chromatographic Fingerprint of traditional Chinese medicine (TCM)” published by China Pharmacopoeia Committee (Version 2012A).

## Results and discussion

### Chemical composition of LBPs

The contents of protein, total carbohydrate, and yield of LBPs, collected from different sites are summarized in Table 1. Each assay was performed in triplicate, the data are expressed as mean ± SD.

**Fig. 1:**
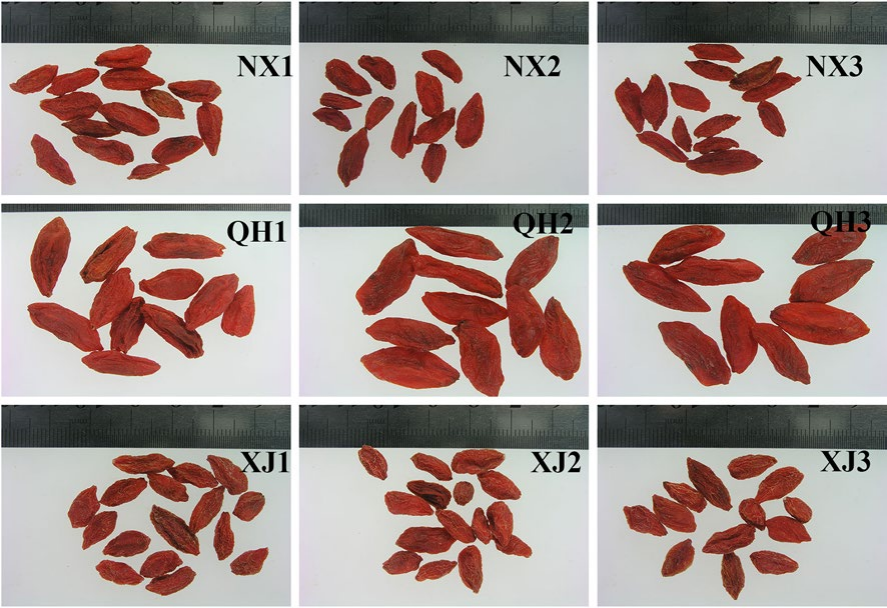
Typical materials of *L. barbarum* collected in China (NX, QH, and XJ *L. barbarum* collected from Ningxia, Qinghai, and Xinjiang in China.)

The average contents of total carbohydrates in the samples from Ningxia, Qinghai, and Xinjiang were 67.4%, 61.9%, and 62.5%, respectively. Our results showed that the average contents of LBPs from Xinjiang, Qinghai and Ningxia were similar. However, the yield of LBPs ranged from 1.6 to 4.8%, indicating that significant differences existed in the yield of LBP’s obtained from plants collected from different origins. The LBPs yields of the plants samples collected from Ningxia and Xinjiang were significantly higher than those from Qinghai (*p* < 0.05), which might have been affected by the specific conditions of the different cultivation regions, such as soil factors [15], light and temperature [16]. This difference is also reflected in the appearance characteristics of the samples (Fig. 1).

The *L. barbarum* fruits collected from Qinghai (average altitude 2740 m, annual average temperature 5.0 °C) had significantly bigger sizes than those from Ningxia (average altitude 1180 m, annual average temperature 10.2 °C) and Xinjiang (average altitude 750 m, annual average temperature 9.5 °C). The results of the determination of 100-grain weight, one of the evaluation indexes utilized in the commercial grade standard for *L. barbarum* evaluation in China were also in agreement with observed appearance characteristics, i.e., the size of the fruits. The average 100-grain weight of three bathes *L. barbarum* fruits from Qinghai (25.59 g) was obviously higher (nearly twice higher) than that of the sample from Ningxia (13.32 g) and Xinjiang (14.29 g). Previous studies revealed that low atmospheric temperature, high altitude, and long sunshine time exerted favorable effects on the appearance characteristics of *L. barbarum* fruits. This conclusion is consistent with our observations. In addition, the *L. barbarum* fruits from Qinghai had the biggest grains, but the yield of LBPs from them was lower than others, which indicated that the yield of LBPs did not increase with the increase of the size of the grain.

### Molecular weight and conformation of LBPs

**Fig. 2:**
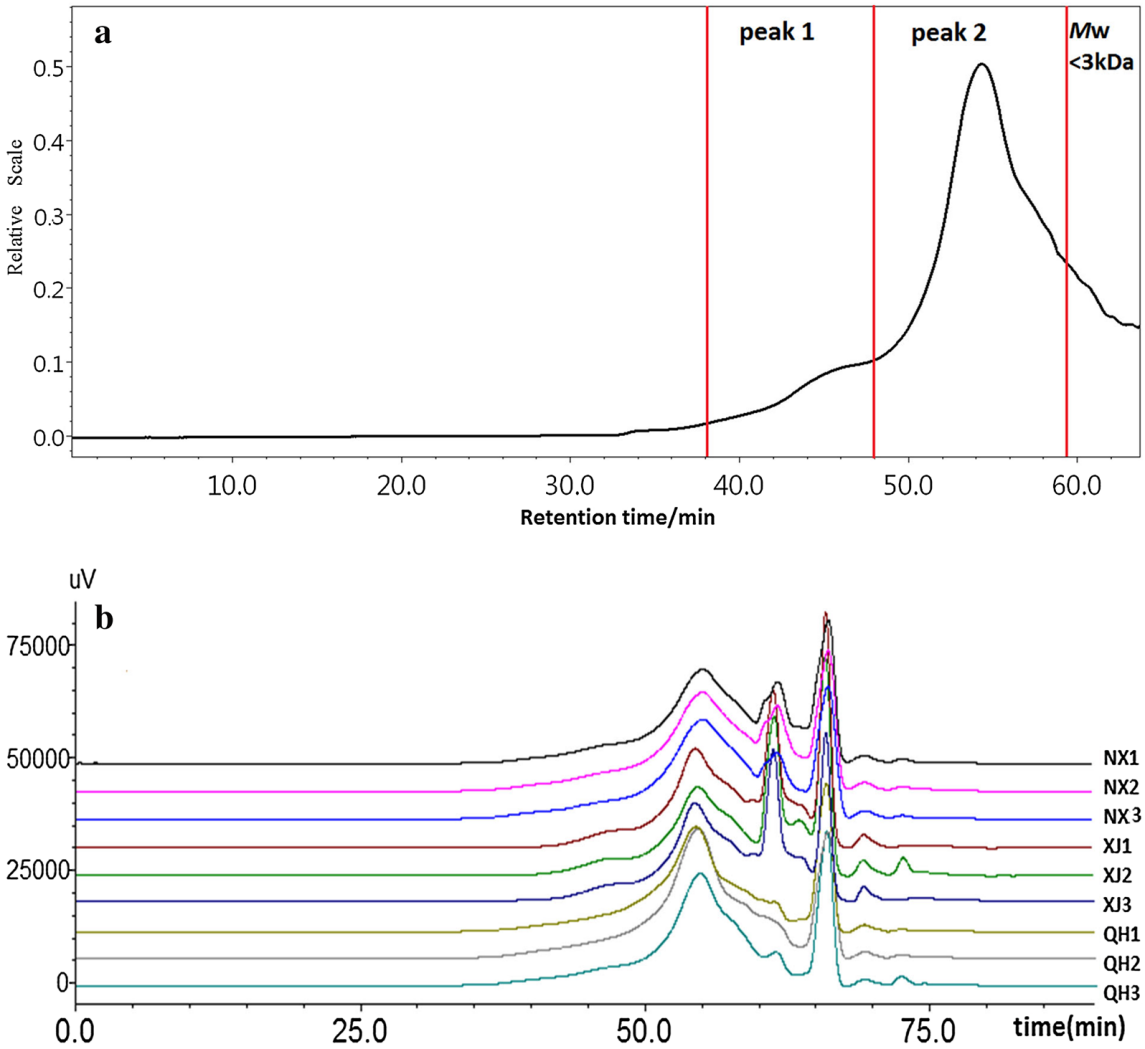
HPSEC chromatograms of LBPs of the samples studied. **a** Typical HPSEC chromatograms of LBPs; **b** comparison among the HPSEC chromatograms of LBPs from the nine batches

Usually, the *M*w of polysaccharides has important effects on their biological activities [17, 18] and is also one of the key indicators to evaluate the quality of polysaccharides. HPSEC-RI-MALLS is an absolute and efficient method to determine the *M*w and polydispersity index of polymers without relying on assumptions regarding column calibration standards or elution properties [19]. To further analyze the structure of LBPs from different areas in China, the HPSEC chromatogram and *M*w were compared. The HPSEC representative chromatogram of LBPs can be seen in Fig. 2b, whereas. Figure 2b represents the HPSEC chromatogram comparison of LBPs of the nine samples examined.

As can be seen in Fig. 2b the HPSEC chromatograms of the nine samples were similar, which implies that the structures of LBPs obtained from different origins are alike. To diminish the possible interference from compounds in our tests, the small-molecular component was partly removed using an ultracentrifugal filter with a molecular weight cutoff value of 3 kDa. The molecular weight (*M*w < 3 kDa) was not precisely determined due to the relatively poor resolution of the column and the co-elution of various different small molecules [6]. Therefore, to increase the accuracy of the determination results, the peak from 59.0 to 64.0 min was not used in the *M*w calculation. The detailed information of the *M*w and polydispersity index (*M*w/*M*n) in LBPs collected from different regions is summarized in Table 2.

**Table 2:** Molecular weight and polydispersity index of LBPs from nine samples

Code	Peak 1		Peak 2		α
	*M*w × 10^6^ Da	*M*w/*M*n	*M*w × 10^4^ Da	*M*w/*M*n	
NX1	1.37 (± 1.4%)	1.58	8.83 (± 1.6%)	1.48	0.233
NX2	1.40 (± 1.2%)	1.64	8.68 (± 1.5%)	1.42	0.197
NX3	1.39 (± 1.3%)	1.56	8.30 (± 1.6%)	1.39	0.251
QH1	1.99 (± 1.2%)	1.66	10.21 (± 1.7%)	1.26	0.189
QH2	1.92 (± 1.0%)	1.76	10.30 (± 1.4%)	1.37	0.205
QH3	2.01 (± 1.0%)	1.92	9.36 (± 1.0%)	1.19	0.197
XJ1	1.46 (± 0.8%)	2.22	6.85 (± 2.7%)	1.38	0.252
XJ2	1.57 (± 0.8%)	2.59	6.97 (± 1.6%)	1.37	0.214
XJ3	1.36 (± 1.8%)	1.76	7.35 (± 2.6%)	1.31	0.243

The results showed that the *M*w of the two polysaccharide fractions (peaks 1–2) in LBPs collected from Ningxia, Qinghai, and Xinjiang were similar, ranging from 1.36 × 10^6^ to 2.01 × 10^6^ (peak 1), and 6.85 × 10^4^ to 10.30 × 10^4^ (peak 2), respectively. These findings were in accordance with the ones of previous studies showing that the molecular weights of polysaccharides in LBPs were within the range from 1.0 × 10^4^ to 2.3 × 10^6^ Da [20]. Furthermore, the polydispersity index of the two polysaccharide fractions (peaks 1 and 2) in LBPs were from 1.56 to 2.59 and from 1.19 to 1.48, respectively, suggesting that molecular the weight distribution of each polysaccharide fraction in *L. barbarum* was relatively narrow.

The polymer conformation of polysaccharides, another important parameter reflecting structure can be determined by HPSEC-MALLS [21]. The conformation, denoted as α, can be examined by determining both *M*w and size (rms radius, rg) at each elution volume, independently, then, plotting log(Rg) as a function of log(*M*w). The resulting slopes reveal whether the molecule’s conformation is approximated by a sphere (slope of about 0.33), random coil (slope of 0.5–0.6) or rod (slope of 1.0) [22]. Of much greater interest was the comparison in molecular conformation, shown in Table 2. The conformation plot, α ranging from 0.189 to 0.252, suggested that the natural LBPs have a compact, sphere-like structure. It also be observed that the conformation of LBPs from nine samples showed high similarity which further demonstrate the consistency of similarity of polysaccharide structure in *L. barbarum.*

### Monosaccharide analysis and HPLC-PAD fingerprints

**Fig. 3:**
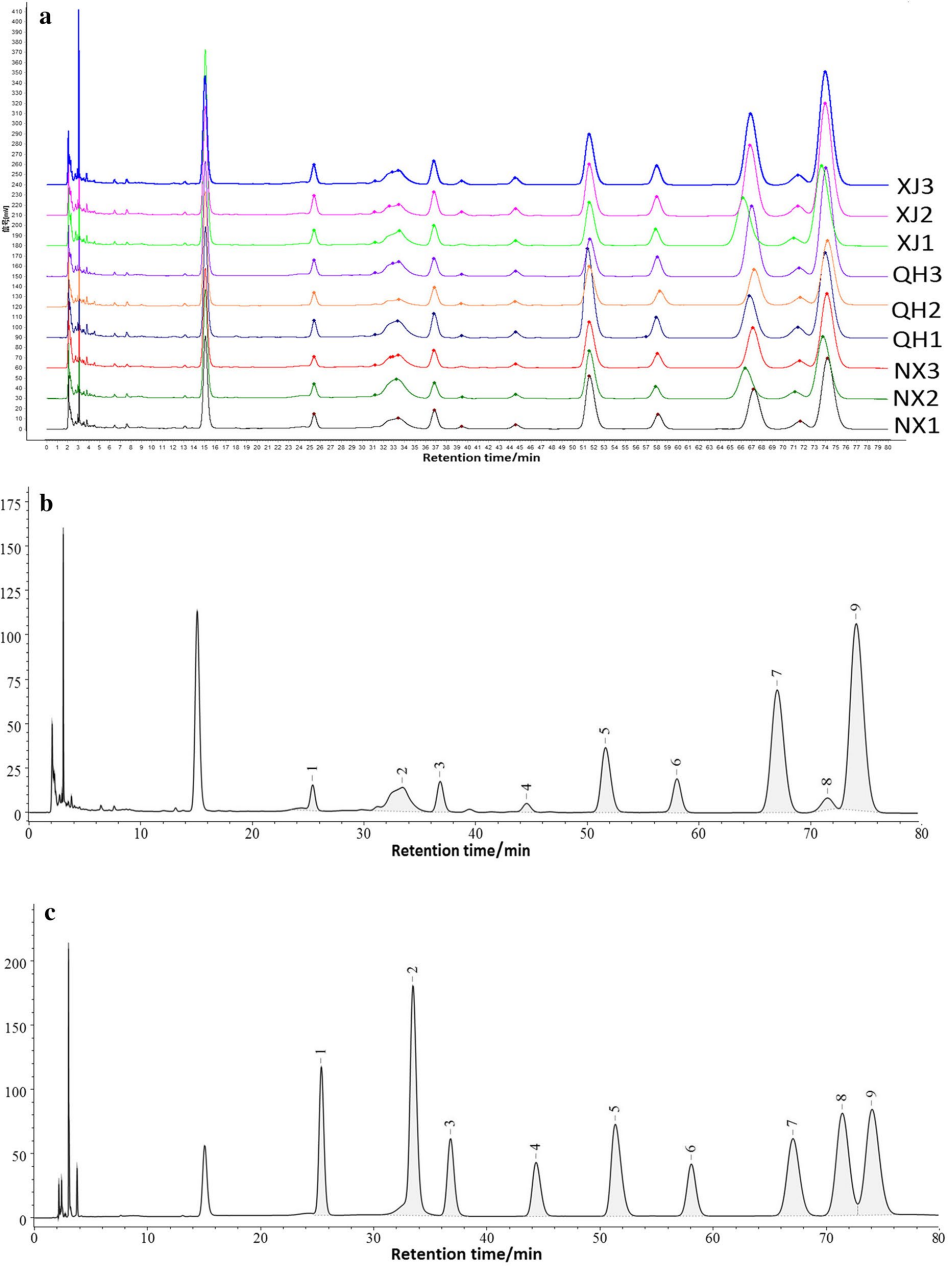
HPLC-PAD chromatogram of LBPs. **a** HPLC-PAD chromatogram of nine batches of LBPs. **b** HPLC-PAD referential fingerprint. **c** HPLC-PAD chromatogram of nine monosaccharide types

The comparison of HPLC-PAD chromatogram from nine samples are presented in Fig. 3a. The results of the HPLC analysis reveal that the LBPs from the different locations consisted of nine types of monosaccharide. The 1st to the 9th peaks represent Man, Rib, Rha, GlcA, GalA, Glc, Gal, Xyl, and Ara. The molar ratios of the monosaccharides in the LBPs of the nine samples are summarized in Table 3, the results in which indicate that the LBPs from different locations had identical monosaccharide compositions, but the molar ratios were not exactly the same.

**Table 3:** Monosaccharide composition of LBPs

Code	Man	Rib	Rha	GlcA	GalA	Glc	Gal	Xyl	Ara
NX1	3.5	3.3	3.8	1.8	26.2	10.0	18.8	2.7	29.9
NX2	4.1	3.7	3.4	2.1	28.7	9.9	16.8	2.7	28.6
NX3	4.2	4.0	3.8	1.8	25.8	10.9	18.4	2.6	28.5
QH1	3.5	3.4	3.2	1.9	22.7	10.9	20.9	2.3	31.3
QH2	4.0	2.6	3.7	2.0	23.4	9.7	20.1	2.7	31.8
QH3	4.2	3.4	4.1	1.8	24.0	9.9	20.3	2.7	29.6
XJ1	4.4	4.1	3.3	1.6	23.4	9.5	20.1	2.4	31.2
XJ2	3.6	3.7	4.0	1.6	25.2	9.6	19.1	2.4	30.7
XJ3	3.5	3.6	4.1	1.5	24.5	11.8	19.1	2.0	30.1
Mean ± SD (n = 9)	3.9 ± 0.37	3.5 ± 0.44	3.7 ± 0.34	1.8 ± 0.19	24.9 ± 1.84	10.2 ± 0.76	19.3 ± 1.23	2.5 ± 0.26	30.2 ± 1.18

With the development of modern analytical technology, extensive exploratory work has been conducted on the fingerprints of polysaccharides from herbs [23, 24]. These studies on polysaccharide fingerprint profiling have often been performed by HPLC. Similarity analysis (SA) have been usually employed to evaluate chromatographic fingerprinting data [25]. In our study, the standard fingerprints were established and the similarity of fingerprints were calculated by the software “Similarity Evaluation System for Chromatographic Fingerprint of Traditional Chinese Medicine (TCM)”. Nine characteristic peaks in fingerprints are displayed in Fig. 3b. The mixed standard sample of nine types of monosaccharide in Fig. 3c was used for qualitative analysis of monosaccharide peaks of samples in the same chromatographic conditions. The results showed that all the similarity values of each of the LBPs with the standard fingerprint were larger than 0.926. The values had highly similar fingerprint characteristics of LBPs although the samples were from different geographical regions. It also implied the standard fingerprint was representative enough that could be used to control and evaluate the quality of *L. barbarum.*

### Antioxidant activities of LBP_S_ from different locations

**Fig. 4:**
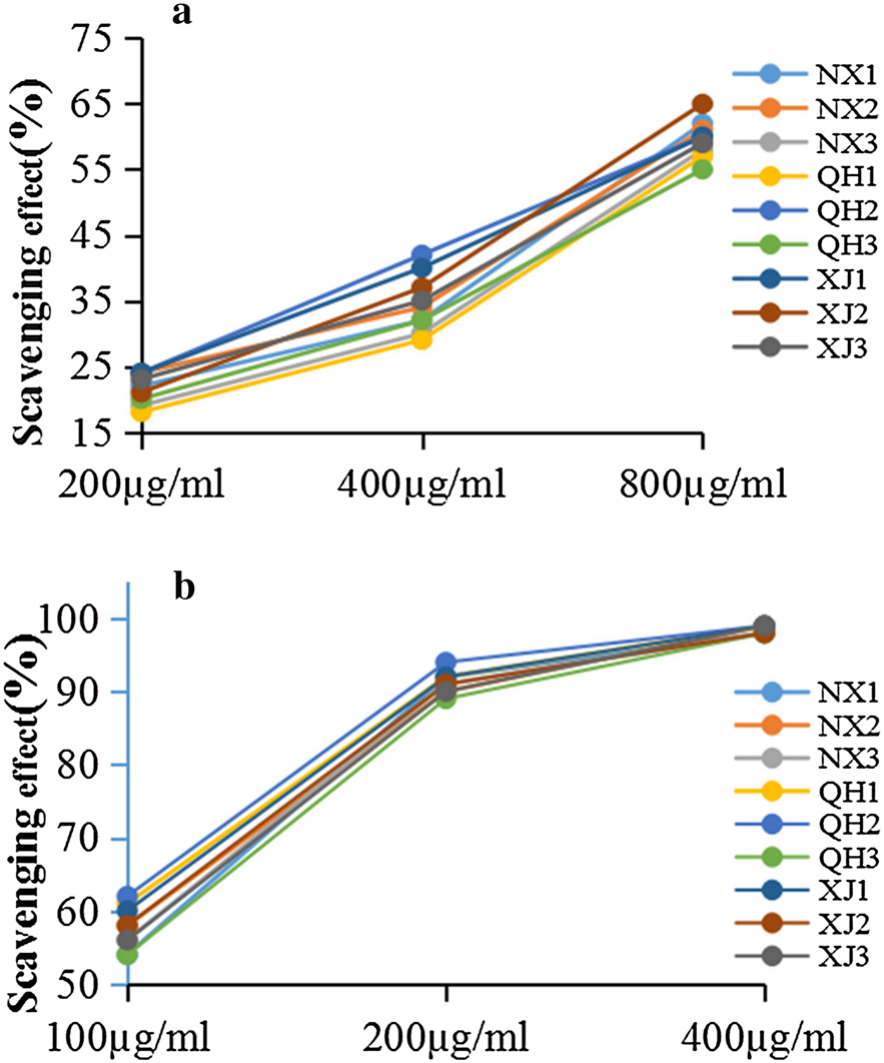
Scavenging effects of nine samples at different concentrations on hydroxyl radicals (**a**) and ABTS radicals (**b**). Each value represents the mean (n = 3)

Two assays were conducted to assess the antioxidant capacities of the nine polysaccharides. The hydroxyl and ABTS radical has been widely used to test the ability of compounds as free radical scavengers or hydrogen donors [26]. The antioxidant activities of LBPs are illustrated in Fig. 4. Our results indicated that the increase in the concentration of polysaccharides enhanced the scavenging activities towards hydroxyl and ABTS radicals. All samples were effective in scavenging hydroxyl radicals, as can be seen in Fig. 4a. The scavenging effect of Ningxia (60.3%, the average value of NX1, NX2 and NX3), Qinghai (57.3%, the average value of QH1, QH2, and QH3) and Xinjiang (61.3%, the average value of XJ1, XJ2 and XJ3) at high concentration (800 µg/mL) was comparable to that of Vit C (95.0%) at 100 µg/mL. The ABTS radical scavenging activities of each sample are illustrated in Fig. 4b. Comparatively, the LBPs at 400 µg/mL concentration possessed a high ABTS radical-scavenging activity (97.7%, the average value of nine samples) which was close to that of V_C_ at low concentration (100 µg/mL).

Our findings indicate that all LBPs from different regions had effective scavenging activities, which may be associated with the structure of LBPs. As mentioned in previous reports [27, 28], polysaccharides is characterized by high contents of galacturonic acid, a high degree of branching and favorable linkage usually showed a strong free radical scavenging activity. Interestingly, LBPs just has these structural features which together contribute to their predominant antioxidant activity. Results also showed that there was no significant difference in antioxidant capacity of nine LBPs towards hydroxyl and ABTS radicals determination.

### Immunomodulatory activities of LBP_S_ from different locations

#### Comparison of RAW264.7 cell viability

**Fig. 5:**
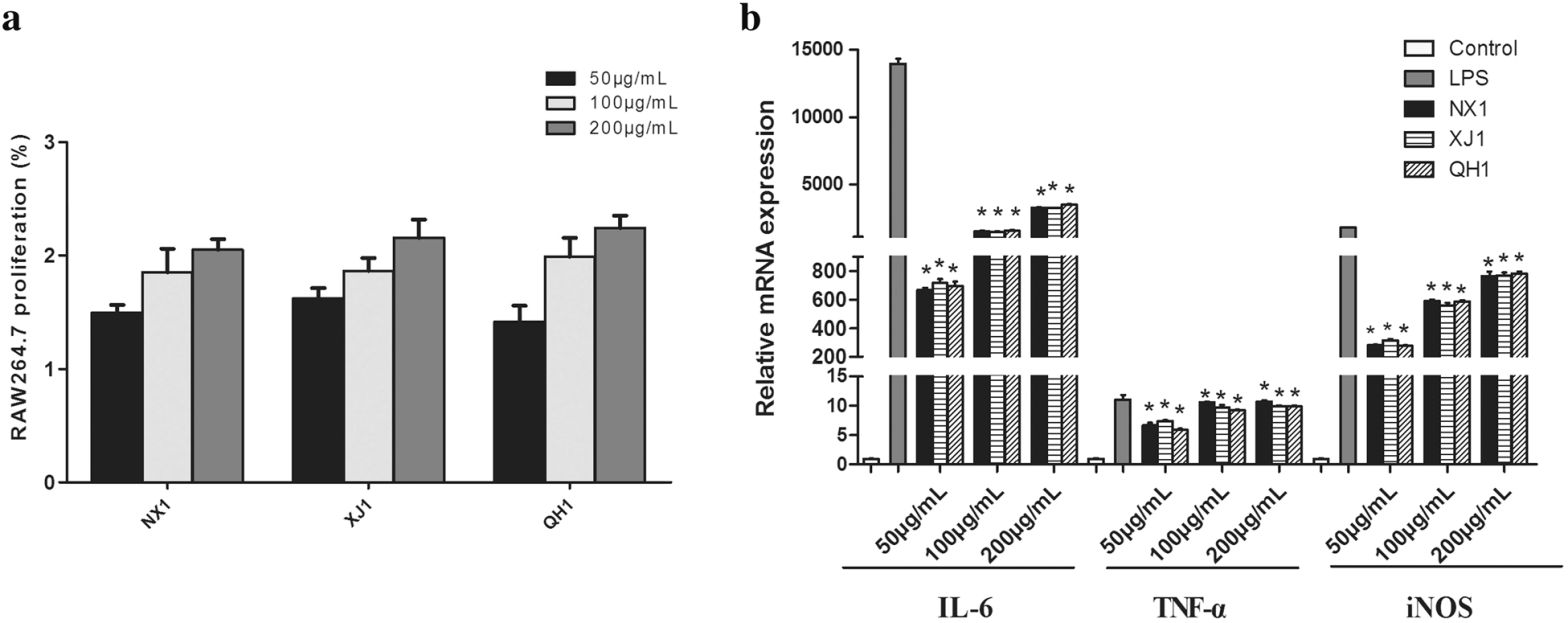
**a** Proliferation activity and **b** mRNA expression of cytokines secretion of NO, IL-6 and TNF-α of RAW264.7 macrophage cells after treatment with polysaccharide from *L. barbarum* (NX1, QH1, XJ1). The values are presented as mean ± SD (n = 3). Significant differences with control cells were designated as *p < 0.05

The cytotoxicity of LBPs from three samples were examined using RAW264.7 macrophage cell lines over the concentration range of 50–200 µg/mL. As presented in Fig. 5a, compared with the untreated cells (control), the viability of macrophage cells incubated with LBPs was more than 100%. When RAW264.7 cells were treated with NX1, significant proliferation up to 150% was observed at 50 µg/mL (p < 0.05). QH1 and XJ1 also stimulate macrophage proliferation up to 160% and 146%. In addition, there was no significant difference in the effect of three LBPs on macrophage proliferation.

#### Effect of LBPs from different locations on RAW264.7 TNF-α, IL-6 and NO production

Regulation of macrophage function by polysaccharides has been reported in diverse ways including promoting macrophage activation, inducing the secretion of TNF**-**α, IL-6 and NO, and enhancing phagocytosis and antigen presenting ability [29, 30]. Thus, the effects of LBPs from different regions induced the secretion of NO, IL-6 and TNF**-**α of macrophage were evaluated and compared in this study. The results showed that RAW264.7 cells treated with NX1, QH1, XJ1 or LPS resulted in significantly increased the mRNA transcription levels of iNOS, IL-6 and TNF-α (Fig. 5b).

## Conclusion

Our results showed that the structural properties including monosaccharide composition, *M*w and conformation of LBPs from different areas were similar, which is consistent with the results obtained from their antioxidant and immune activities. It may implied that the species is a more important factor affecting the structure and bioactivities of LBPs than the origin. However, the average yield of LBPs from different producing areas showed great differences: the LBPs from Qinghai were significantly lower (p < 0.05) than those from Xinjiang and Ningxia, which may indicate that the *L. barbarum* produced in Ningxia and Xinjiang maybe more suitable as materials for medicines and functional foods.

This study also provides a reference for improving the quality control standard of LBPs. According to our research results, in consideration of structural similarity of LBPs from different producing areas, the yield of LBPs should be used as a key index to evaluate the quality of *L. barbarum*. First, the HPLC-PAD typical fingerprint of the monosaccharide composition and *M*w of peaks can be determined to identify the LBPs and the related products from polysaccharides of other herbs. Then the yield of LBPs can be used as an important index to evaluate the quality of *L. barbarum*. On the basis of the existing standards for the determination of LBPs content, the identification tests for the determination of monosaccharide composition and *M*w should also be developed. Nevertheless, conducting a large-scale study, including the analysis of larger numbers of samples from Ningxia, Xinjiang, and Qinghai, is required to further confirm the present results.

## Data Availability

Not applicable.

## References

[1] Amagase H, Sun B, Borek C (2009). *Lycium barbarum* (goji) juice improves in vivo antioxidant biomarkers in serum of healthy adults. Nutr Res.

[2] Gan L, Hua Zhang S, Liang Yang X, Bi XuH (2004). Immunomodulation and antitumor activity by a polysaccharide-protein complex from *Lycium barbarum*. Int Immunopharmacol.

[3] Yao X, Li-Jia XU, Xiao W, Peng Y, Xiao PG (2011). Analysis of *Lycium barbarum* polysaccharide from different lycii fructus. Herald Med.

[4] Cuesta G, Suarez N, Bessio MI, Ferreira F, Massaldi H (2003). Quantitative determination of pneumococcal capsular polysaccharide serotype 14 using a modification of phenol–sulfuric acid method. J Microbiol Methods.

[5] Liu W, Xu J, Zhu R, Zhu Y, Zhao Y, Chen P (2015). Fingerprinting profile of polysaccharides from *Lycium barbarum* using multiplex approaches and chemometrics. Int J Biol Macromol.

[6] Wu DT, Lam SC, Cheong KL, Wei F, Lin PC, Long ZR (2016). Simultaneous determination of molecular weights and contents of water-soluble polysaccharides and their fractions from *Lycium barbarum* collected in China. J Pharm Biomed Anal.

[7] Lin CL, Wang CC, Chang SC, Inbaraj BS, Chen BH (2009). Antioxidative activity of polysaccharide fractions isolated from *Lycium barbarum* Linnaeus. Int J Biol Macromol.

[8] Zhao Q, Dong B, Chen J, Zhao B, Wang X, Wang L (2015). Effect of drying methods on physicochemical properties and antioxidant activities of wolfberry (*Lycium barbarum*) polysaccharide. Carbohyd Polym.

[9] Xie J, Wu DT, Li WZ, Ning CG, Tang YP, Zhao J (2017). Effects of polysaccharides in lycium barbarum berries from different regions of China on macrophages function and their correlation to the glycosidic linkages. J Food Sci.

[10] Redgwell RJ, Curti D, Wang J, Dobruchowska JM, Gerwig GJ, Kamerling JP (2011). Cell wall polysaccharides of Chinese Wolfberry (*Lycium barbarum*): Part 1. Characterisation of soluble and insoluble polymer fractions. Carbohydr Polym.

[11] Zhu J, Liu W, Yu J, Zou S, Wang J, Yao W (2013). Characterization and hypoglycemic effect of a polysaccharide extracted from the fruit of *Lycium barbarum* L. Carbohydr Polym.

[12] Wu WL, Zhu YT, Zhang L, Yang RW, Zhou YH (2012). Extraction, preliminary structural characterization, and antioxidant activities of polysaccharides from *Salvia miltiorrhiza* Bunge. Carbohydr Polym.

[13] Chen Y, Li XH, Zhou LY, Li W, Liu L, Wang DD (2017). Structural elucidation of three antioxidative polysaccharides from *Tricholoma lobayense*. Carbohydr Polym.

[14] Memarpoor-Yazdi M, Asoodeh A, Chamani J (2012). A novel antioxidant and antimicrobial peptide from hen egg white lysozyme hydrolysates. J Funct Foods.

[15] Aruani MC, Reeb PD, Barnes NE (2014). Influence of soil properties on yield and fruit maturity at harvest of ‘williams’ pear. Chil J Agric Res.

[16] Medlicott AP, Reynolds SB, Thompson AK (2010). Effects of temperature on the ripening of mango fruit (*Mangifera indica* L. var. tommy atkins). J Sci Food Agric.

[17] Sun L, Ling W, Yan Z (2012). Immunomodulation and antitumor activities of different-molecular-weight polysaccharides from *Porphyridium cruentum*. Carbohydr Polym.

[18] Sheng J, Sun Y (2014). Antioxidant properties of different molecular weight polysaccharides from *Athyrium multidentatum* (Doll.) Ching. Carbohydr Polym.

[19] Hu DJ, Cheong KL, Zhao J, Li SP (2013). Chromatography in characterization of polysaccharides from medicinal plants and fungi. J Sep Sci.

[20] Liang B, Jin M, Liu H (2011). Water-soluble polysaccharide from dried *Lycium barbarum* fruits: isolation, structural features and antioxidant activity. Carbohydr Polym.

[21] Cheong KL, Wu DT, Zhao J, Li SP (2015). A rapid and accurate method for the quantitative estimation of natural polysaccharides and their fractions using high performance size exclusion chromatography coupled with multi-angle laser light scattering and refractive index detector. J Chromatogr A.

[22] Liu W, Liu Y, Zhu R, Yu J, Lu W, Pan C (2016). Structure characterization, chemical and enzymatic degradation, and chain conformation of an acidic polysaccharide from *Lycium barbarum* L. Carbohydr Polym.

[23] Wang Y, Xian J, Xi X, Wei X (2013). Multi-fingerprint and quality control analysis of tea polysaccharides. Carbohydr Polym.

[24] Sun X, Wang H, Han X, Chen S, Zhu S, Dai J (2014). Fingerprint analysis of polysaccharides from different Ganoderma by HPLC combined with chemometrics methods. Carbohydr Polym.

[25] Yang Z, Niu Y, Xie Z, Shi H, Pei C, Yu L (2013). Differentiating leaf and whole-plant samples of di- and tetraploid *Gynostemma pentaphyllum* (Thunb.) Makino using flow-injection mass spectrometric fingerprinting method. J Funct Foods.

[26] Jao CL, Wen-Ching KO (2011). 1,1Diphenyl2-picrylhydrazyl (DPPH) radical scavenging by protein hydrolyzates from tuna cooking juice. Fish Sci.

[27] Asker MMS, Mahmoud MG, Ibrahim GS (2007). Structural characterization and biological activity of acidic polysaccharide fractions isolated from *Bacillus polymyxa* NRC-A. J Appl Sci Res.

[28] Volman JJ, Helsper JP, Wei S, Baars JJ (2010). Effects of mushroom-derived beta-glucan-rich polysaccharide extracts on nitric oxide production by bone marrow-derived macrophages and nuclear factor-kappaB transactivation in Caco-2 reporter cells: can effects be explained by structure?. Mol Nutr Food Res.

[29] Mehdi T, SangGuan Y, Elham HD, Utoomporn S (2018). Water-soluble polysaccharides from *Ulva intestinalis*: molecular properties, structural elucidation and immunomodulatory activities. J Food Drug Anal.

[30] Zhang X, Li Y, Cheng J, Liu G, Qi C, Zhou W (2014). Immune activities comparison of polysaccharide and polysaccharide-protein complex from *Lycium barbarum* L. Int J Biol Macromol.

